# miRNAs in lung cancer - Studying complex fingerprints in patient's blood cells by microarray experiments

**DOI:** 10.1186/1471-2407-9-353

**Published:** 2009-10-06

**Authors:** Andreas Keller, Petra Leidinger, Anne Borries, Anke Wendschlag, Frank Wucherpfennig, Matthias Scheffler, Hanno Huwer, Hans-Peter Lenhof, Eckart Meese

**Affiliations:** 1 febit biomed gmbh, Im Neuenheimer Feld 519, 69120 Heidelberg, Germany; 2 Department of Human Genetics, Medical School, Saarland University, Building 60, 66421 Homburg, Germany; 3 Department of Cardiothoracic Surgery, Voelklingen Heart Center, 66333 Voelklingen, Germany; 4 Center for Bioinformatics, Saarland University, Building E 1 1, 66041Saarbruecken, Germany

## Abstract

**Background:**

Deregulated miRNAs are found in cancer cells and recently in blood cells of cancer patients. Due to their inherent stability miRNAs may offer themselves for blood based tumor diagnosis. Here we addressed the question whether there is a sufficient number of miRNAs deregulated in blood cells of cancer patients to be able to distinguish between cancer patients and controls.

**Methods:**

We synthesized 866 human miRNAs and miRNA star sequences as annotated in the Sanger miRBase onto a microarray designed by febit biomed gmbh. Using the fully automated Geniom Real Time Analyzer platform, we analyzed the miRNA expression in 17 blood cell samples of patients with non-small cell lung carcinomas (NSCLC) and in 19 blood samples of healthy controls.

**Results:**

Using t-test, we detected 27 miRNAs significantly deregulated in blood cells of lung cancer patients as compared to the controls. Some of these miRNAs were validated using qRT-PCR. To estimate the value of each deregulated miRNA, we grouped all miRNAs according to their diagnostic information that was measured by Mutual Information. Using a subset of 24 miRNAs, a radial basis function Support Vector Machine allowed for discriminating between blood cellsamples of tumor patients and controls with an accuracy of 95.4% [94.9%-95.9%], a specificity of 98.1% [97.3%-98.8%], and a sensitivity of 92.5% [91.8%-92.5%].

**Conclusion:**

Our findings support the idea that neoplasia may lead to a deregulation of miRNA expression in blood cells of cancer patients compared to blood cells of healthy individuals. Furthermore, we provide evidence that miRNA patterns can be used to detect human cancers from blood cells.

## Background

Lung cancer is the leading cause of cancer death worldwide [1]. Its five-year survival rate is among the lowest of all cancer types and is markedly correlated to the stage at the time of diagnosis [2]. Using currently existing techniques, more than two-thirds of lung cancers are diagnosed at late stages, when the relative survival rate is low [3]. This reality calls for the search of new biomarkers that are able to catch lung cancer while it is still small and locally defined.

MicroRNAs (miRNA) are a recently discovered class of small non-coding RNAs (17-24 nucleotides) [4]. Due to their function as regulators of gene expression they play a critical role both in physiological and in pathological processes, such as cancer [5-8]. This fact is also outlined by the "Human MiRNAs & Diseases" database, the most comprehensive resource on the web, containing hundreds of entries showing the deregulation of miRNAs in a manifold of human diseases [9].

There is increasing evidence that microRNAs are not only found in tissues but also in human blood cells both as free circulating nucleic acids and in mononuclear cells. A recent proof-of-principle study demonstrated miRNA expression pattern in pooled blood sera and pooled blood cells, both in healthy individuals and in cancer patients including patients with lung cancer [10]. In addition, a remarkable stability of miRNAs in human sera was recently demonstrated [10,11]. These findings make miRNA a potential tool for a cancer diagnostics based on blood analysis. Since single biomarkers usually lack sufficient specificity and sensitivity, we set out to analyze complex miRNA expression pattern in blood cells of cancer patients. We synthesized 866 human miRNAs and miRNA star sequences as annotated in the Sanger miRBase ([12,13], Version 12.0) on a microarray designed by febit biomed gmbh. This array combined with the fully automated Geniom Real Time Analyzer (GRTA) platform allows for measuring miRNA fingerprints and ensures a high degree of reproducibility. To identify miRNA expression pattern we analyzed the expression of 866 miRNAs in 17 blood samples of patients with non-small cell lung carcinomas and in 19 blood samples of healthy controls.

The aim of our study was to address the following questions: Is there a larger number of differentially regulated miRNAs in blood cells of lung cancer patients as compared to healthy controls? To what extend do miRNA expression profiles in blood cells allow for the discrimination of lung cancer patients from controls? What is the information content of single miRNAs for such discrimination? Does the platform used in these experiments offer the option of a highly reproducible and efficient large-scale diagnostic test? The answers to these questions will also lay the ground for the analysis of blood based miRNA expression profiles in other cancers.

## Methods

### Samples

The analysis of blood from lung cancer patients and healthy subjects has been approved by local ethics committee and participants have given written informed consent. Blood samples were obtained with patients' informed consent. The patient samples stem from 17 patients with non-small cell lung carcinoma and normal controls. Normal samples were obtained from 19 different volunteers. More detailed information of patients and controls is given in Tables 1 and 2.

**Table 1:** Information on lung cancer patients and healthy control subjects

blood donors	male	female
**lung cancer patients**		
number	9	8
average age	67	61
		
**healthy subjects**		
number	7	12
average age	43	35

**Table 2:** Staging of patients and tumor type

sample	tumor type	clinical stage
463	adenocarcinoma	T2N0
464	typical carcinoid	T2N0
468	adenocarcinoma	T2N0
469	adenocarcinoma	T2N2
485	adenocarcinoma	T2N0
490	adeno squamous carcinoma	T2N0
492	adenocarcinoma	T2N0
503	squamous cell carcinoma	T1N0
507	squamous cell carcinoma	T4Nx
508	squamous cell carcinoma	T2aN0
509	squamous cell carcinoma	T2N0
513	bronchoalveolar carcinoma	T3N0
514	adenocarcinoma	T2N2
517	squamous cell carcinoma	T2N0
523	squamous cell carcinoma	T2N0
524	squamous cell carcinoma	T3N2
525	adenocarcinoma	T1N0

### miRNA extraction and microarray screening

Blood of lung cancer patients and volunteers without known disease was extracted in PAXgene Blood RNA tubes (BD, Franklin Lakes, New Jersey USA). For each blood donor, 5 ml of peripheral blood were obtained. The content of blood sampling tubes of each patient and healthy donor was centrifuged at 5000 g for 10 min at room temperature. The supernatant was discarded while the pellet was resuspended in 10 ml RNase free water by vortex. The suspension is again centrifuged at 5000 g for 10 min at RT. Subsequently, the total RNA including the miRNA was isolated using the miRNeasy kit (Qiagen GmbH, Hilden). Therefore the pellet was resuspended in 700 μl QIAzol lysis reagent and incubated for 5 min at RT. Then, 140 μl chloroform were added, vortexed for 15 sec, and incubated for 2-3 min at RT. This was followed by a centrifugation at 14000 rpm and 4°C for 15 min. The upper, watery phase was removed and 1,5 times of its volume added in 100% ethanol (ca. 900 μl). Each 700 μl of this mixture were placed on a column and centrifuged at 13000 rpm at RT for 15 sec. After the mixture had completely passed the column, 700 μl of Buffer RWT were added to each column, and again centrifuged at 13000 rpm at RT for 15 sec. Following this, 500 μl Buffer RPL were added to the column and centrifuged at 13000 rpm at RT for 15 sec. After this, another 500 μl Buffer RPL were added to the column and centrifuged at 13000 rpm at RT for 2 min. The column was then centrifuged at 13000 rpm and RT for 1 min to dry it. The following was the elution step with 40 μl RNase free water, centrifuging at 13000 rpm at RT for 1 min. The eluted RNA was stored at -70°C.

Samples were analyzed with the Geniom Realtime Analyzer (GRTA, febit gmbh, Heidelberg, Germany) using the Geniom Biochip miRNA homo sapiens. Each array contains 7 replicates of 866 miRNAs and miRNA star sequences as annotated in the Sanger miRBase 12.0 [13,14]. Sample labeling with Biotine has been carried out either by using the miRVANA™ miRNA Labeling Kit (Applied Biosystems Inc, Foster City, California USA) or by microfluidic-based enzymatic on-chip labeling of miRNAs (MPEA [15]).

Following hybridization for 16 hours at 42°C the biochip was washed automatically and a program for signal enhancement was processed with the GRTA. The resulting detection pictures were evaluated using the Geniom Wizard Software. For each array, the median signal intensity was extracted from the raw data file such that for each miRNA seven intensity values have been calculated corresponding to each replicate copy of miRBase on the array. Following background correction, the seven replicate intensity values of each miRNA were summarized by their median value. To normalize the data across different arrays, quantile normalization [16] was applied and all further analyses were carried out using the normalized and background subtracted intensity values.

We stored all microarray data in the miRDBXP (manuscript in preparation), a database which is designed to store any type of microRNA expression pattern. The database is freely accessible at http://64.119.137.93/fmi/iwp. In addition, the data are also available in GEO (GSE17681).

### Statistical analysis

After having verified the normal distribution of the measured data, we carried out parametric t-test (unpaired, two-tailed) for each miRNA separately, to detect miRNAs that show a different behavior in different groups of blood donors. The resulting p-values were adjusted for multiple testing by Benjamini-Hochberg [17,18] adjustment. Moreover, the Mutual Information (MI) [19] was computed as a measure to access the diagnostic value of single miRNA biomarkers. To this end, all biomarkers were transformed to z-scores and binned in three bins before the MI values of each biomarker, and the information whether the marker has been measured from a normal or lung cancer sample, was computed. In addition to the single biomarker analysis classification of samples using miRNA patterns was carried out using Support Vector Machines (SVM, [20]) as implemented in the R [21] e1071 package. In detail, different kernel (linear, polynomial, sigmoid, radial basis function) Support Vector Machines have been evaluated, where the cost parameter was sampled from 0.01 to 10 in decimal powers. The measured miRNA profiles were classified using 100 repetitions of standard 10-fold cross-validation. As a subset selection technique we applied a filter approach based on t-test. In detail, the *s *miRNAs with lowest p-values were computed on the training set in each fold of the cross validation, where *s *was sampled from 1 to 866. The respective subset was used to train the SVM and to carry out the prediction of the test samples. As result, the mean accuracy, specificity, and sensitivity were calculated together with the 95% Confidence Intervals (95% CI) for each subset size. To check for overtraining we applied permutation tests. Here we sampled the class labels randomly and carried out classifications using the permuted class labels. All statistical analyzes were performed using R [21-23].

## Results

### miRNA experiments

We analyzed the expression of 866 miRNAs and miRNA star sequences in blood cells of 17 patients with NSCLC. As a control we used blood cells of 19 volunteers without known disease (see also Materials and Methods).

Following RNA isolation and labeling by miRVANA™ miRNA Labeling Kit, the miRNA expression profiles were measured by the Geniom Bioship miRNA homo sapiens in the GRTA (febit gmbh, Heidelberg). Following intensity value computation and quantile normalization of the miRNA profiles [16], we determined a mean correlation value of 0.97 for technical replicates by using purchased total RNA from Ambion (four heart and four liver replicates). As comparison, we computed the mean correlation of all cancer samples with all other cancer samples and all normal samples with all other normal samples. Here, we still reached a high correlation of 0.87 and a variance of 0.009.

### ruling out the influence of age and gender

To cross-check that age and gender do not have an influence on our analysis, we computed t-tests for the normal samples. In the case of males versus females we did not find any statistically significant deregulated miRNA. The most significant miRNA, hsa-miR-423, showed an adjusted significance level of 0.78.

To test for the influence of donor age we compared the profiles obtained from samples obtains from the oldest versus youngest patients by splitting the group in half based on age. Here the most significant miRNA, miR-890, obtained an adjusted p-value of 0.87. As for gender, we did not find any deregulated miRNAs, thus providing evidence that age and gender do not have a substantial influence on the miRNA profiles.

### single deregulated miRNAs

**Figure 1:**
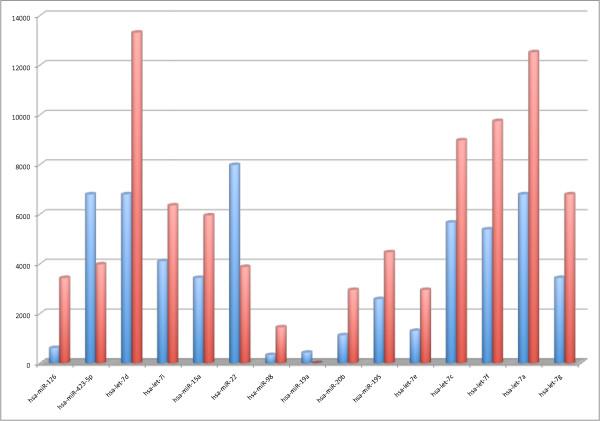
**The bar-chart shows for 15 of the 27 deregulated miRNAs the median value of cancer samples and normal samples**. Here, blue bars correspond to cancer samples while red bars to controls.

**Figure 2:**
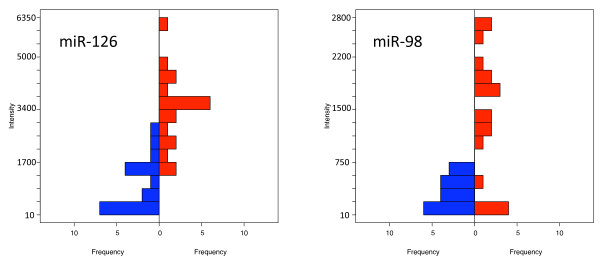
**Back to back histograms of two examples**. The color-coding corresponds to the one used in Figure 1.

We applied hypothesis testing to identify miRNAs deregulated in the blood cells of lung cancer patients as compared to the blood cells of the controls. Following verification of an approximately normal distribution, we performed two-tailed unpaired t-tests for each miRNA. The respective p-values were adjusted for multiple testing by the Benjamini-Hochberg approach [17,18]. In total we detected 27 miRNAs significantly deregulated in blood cells of lung cancer patients as compared to the controls. A complete list of deregulated miRNAs is given in the Supplemental Material (see Additional file 1 - Table S1). The miRNAs that were most significantly deregulated included hsa-miR-126 with a p-value of 0.00003, hsa-let-7d with a p-value of 0.003, hsa-let-7i with a p-value of 0.003, and hsa-miR-423 with a p-value of 0.001 (Figure 1 and Figure 2). Other members of the let-7 family that were also found to be deregulated included hsa-let-7c, hsa-let-7e, hsa-let-7f, hsa-let-7g and hsa-let-7a. Besides miR-423, all above mentioned miRNAs were down-regulated in blood cells of lung cancer patients compared to blood cells of healthy subjects indicating an overall decreased miRNA repertoire.

To validate our findings, we repeated the miRNA profiling using an enzymatic on-chip labeling technique termed MPEA (Microfluidic-based enzymatic on-chip labeling of miRNAs) [24]. For this control experiment, we used 4 out of the 17 lung cancer patients and 10 of the controls. Hereby, we detected 100 differentially regulated miRNAs. The miRNAs that were most significantly deregulated include hsa-miR-1253 with a p-value of 0.001, hsa-miR-126 with a p-value of 0.006, hsa-let-7d with a p-value of 0.006, and hsa-let-7f with a p-value of 0.006. Of the previously identified 27 miRNAs 12 were detected to be significant in the second experiment, while the remaining miRNAs showed increased p-values. The correlation of fold changes was 0.62. We also confirmed other members of the let-7 family as deregulated in blood cells of lung cancer patients. Furthermore, we confirmed that the majority of the deregulated miRNAs were down-regulated in patients' blood cells. In these Experiments, 62% of the significantly deregulated miRNAs showed decreased intensity values in lung cancer samples. In the Supplemental Material we provide a complete list of deregulated miRNAs identified by MPEA (see Additional file 1 - Table S1).

**Figure 3:**
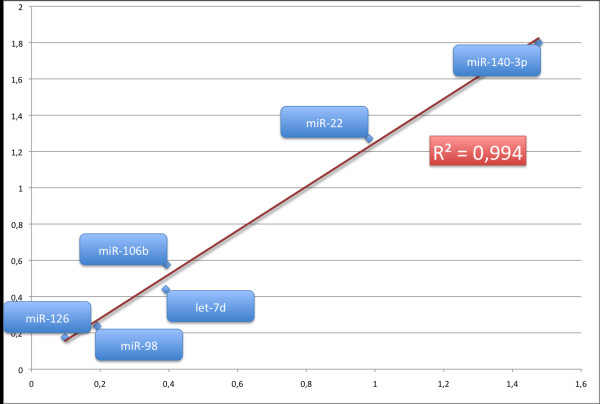
**Scatterplot of fold quotients of qRT-PCR (x-axis) and microarray experiments (y-axis)**.

As a further control experiment we performed an expression analysis by qRT-PCR. As a test sample we analyzed the fold changes of hsa-miR-106b, miR-162, miR-98, miR-let7d, miR-22, and miR-140-3p in blood cells of eight tumor patients and five controls. Of these 6 miRNAs, 3 belong to the group of 12 miRNAs significant for miRVANA labeling and the MPEA method. The result of this validation experiment was a verification of the microarray screening. For example, in agreement with the up-regulation detected by the Geniom Biochip experiments, the qRT-PCR experiments showed on average an up-regulation of 1.4 fold in blood cells of lung cancer patients as compared to healthy controls for miR 106b. The excellent correlation of microarray and qRT-PCR data is demonstrated by an R^2 ^value of 0.994 (see Figure 3).

### Diagnostic value of miRNA biomarkers

Mutual Information (MI) [19] is an adequate measure to estimate the overall diagnostic information content of single biomarkers [22]. In our study, Mutual Information is considered as the reduction in uncertainty about the class labels '0' for controls and '1' for tumor samples due to the knowledge of the miRNA expression. The higher the value of the MI of a miRNA, the higher is the diagnostic content of the respective miRNA.

**Figure 4:**
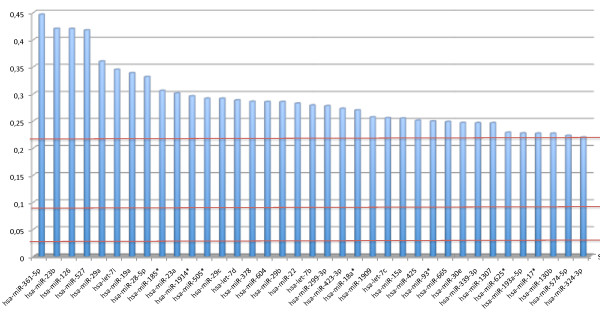
**The mutual information of all miRNAs that have higher information content than the best permutation test (upper red line)**. The middle red line denotes the 95% quantile of the 1000 permutation tests and the bottom red line the mean of the permutation experiments, corresponding to the background MI.

**Figure 5:**
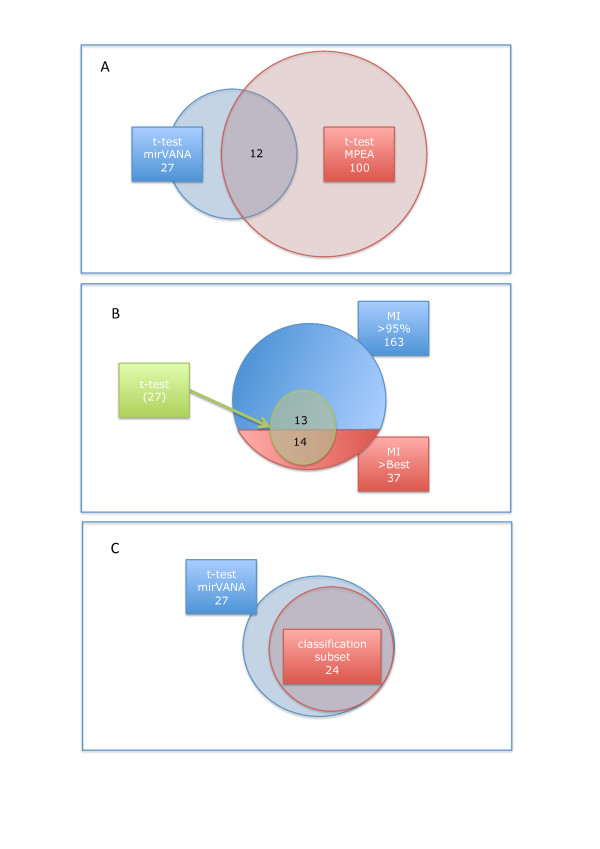
**Venn diagrams for different analyses**. A) Venn diagram of the t-test mirVANA and MPEA labeling. B) Venn diagram of the t-test and the Mutual Information. The right circle shows significant MI miRNAs, separated in the miRNAs that are higher than the highest permutation test and the miRNAs higher than 95% of all 1000 permutation tests. The 27 miRNAs are all included in these miRNAs, indicated by the green circle. C) Venn diagram of t-test miRNAs and miRNAs used in the best classification subset. The 24 miRNAs used for classification are all contained in the set of 27 most significant mRNAs

We computed the MI of each miRNA with the class labels. First, we carried out a permutation test to determine the background noise of the miRNAs, e.g. the random information content of each miRNA. We randomly selected 1000 miRNAs (with replacements) and sampled the class labels for each miRNA. These permutation tests yielded a mean MI value of 0.029, a 95% quantile of 0.096 and a value of 0.217 for the highest random MI. Second, we calculated the MI values for the comparison between the miRNAs in blood cells of tumor patients and controls. The overall comparison of the 866 miRNAs yielded significantly increased MI values with a two-tailed p-value of ≤ 10^-10 ^as shown by an unpaired Wilcoxon Mann-Whitney test [23,24]. The miRNA hsa-miR-361-5p showed the highest MI with a value of 0.446. The miRNAs with the best significance values as computed by the t-test, namely hsa-miR-126 and hsa-miR-98, were also among the miRNAs showing the highest MI values. In total we detected 37 miRNAs with MI values higher than the highest of 1000 permuted miRNAs and 200 miRNAs with MI values higher than the 95% quantile (Figure 4). Of the initially detected 27 miRNAs by the t-test, 14 had values higher than the highest permutation test and all remaining 13 miRNAs belong to the group of miRNAs with MI values higher than the 95% quantile, demonstrating the overall well agreement of the two measures. Venn diagrams for the different analyses are provided as Venn diagrams (Figures 5A and 5B). A complete list of miRNAs, the respective MI and the enrichment compared to the background MI is provided in the supplemental material (see Additional file 2 - Table S2).

### Evaluating complex fingerprints

Even single miRNAs with highest MI values are not sufficient to differentiate between blood cells of tumor patients as compared to controls with high specificity. For example, the hsa-miR-126 separates blood cells of tumor patients from blood cells of healthy individuals with a specificity of 68%, only. In order to improve the classification accuracy we combined the predictive power of multiple miRNAs by using statistical learning techniques. In detail, we applied Support Vector Machines with different kernels (linear, polynomial, sigmoid, radial basis function) to the data and carried out a hypothesis test based subset selection as described in Material and Methods. To gain statistical significance we carried out 100 repetitions of 10-fold cross validation. Likewise, we computed 100 repetitions for the permutation tests.

**Figure 6:**
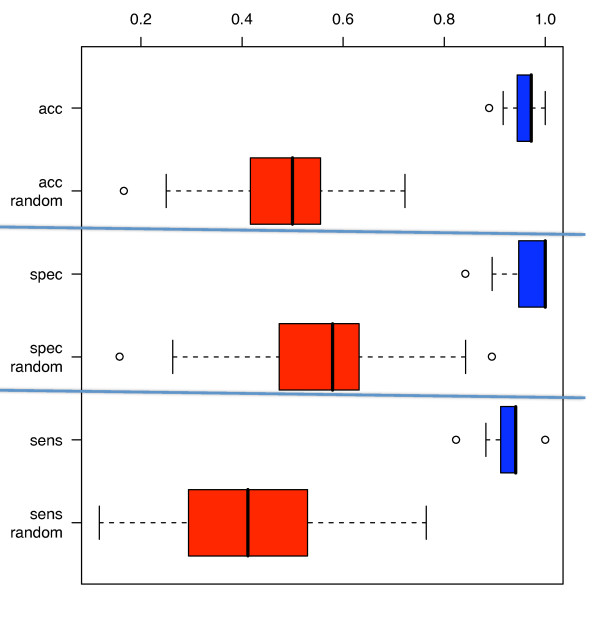
**Boxplots of the classification accuracy, specificity, and sensitivity of the 100 repetitions of the 10-fold cross-validation (red boxes) and the same values for random permutation tests (blue boxes)**.

The best results were obtained with radial basis function Support Vector Machines and a subset of 24 miRNAs (Table 3 and Venn Diagram in Figure 5C). These miRNAs allowed for the discrimination between blood cells of lung tumor patients and blood cells of controls with an accuracy of 95.4% [94.9%-95.9%], a specificity of 98.1% [97.3%-98.8%], and a sensitivity of 92.5% [91.8%-92.5%]. The permutation tests showed significantly decreased accuracy, specificity, and sensitivity with 49.2% [47.2%-51.3%], 56.9% [54.5%-59.3%] and 40.6% [37.9%-43.4%], respectively (Figure 6), providing evidence that the obtained results are not due to an overfit of the statistical model on the miRNA fingerprints.

**Table 3:** miRNAs that are in the best subset

miRNA	median cancer	median control	Fold change	Log(FC)	t-test adj
hsa-miR-126	606,46	3428,42	0,18	-1,73	3,43E-05
hsa-miR-423-5p	6795,89	3976,97	1,71	0,54	0,000978034
hsa-let-7d	6795,89	13307,74	0,51	-0,67	0,002715951
hsa-let-7i	4106,11	6349,31	0,65	-0,44	0,002715951
hsa-miR-15a	3428,42	5944,79	0,58	-0,55	0,008784487
hsa-miR-22	7978,5	3868,5	2,06	0,72	0,008784487
hsa-miR-98	322,44	1440,75	0,22	-1,5	0,008784487
hsa-miR-19a	420,06	1	420,06	6,04	0,015204977
hsa-miR-20b	1118,35	2947,83	0,38	-0,97	0,015204977
hsa-miR-324-3p	1221,94	700,5	1,74	0,56	0,015204977
hsa-miR-574-5p	108,56	30,22	3,59	1,28	0,015204977
hsa-miR-195	2575,72	4462,58	0,58	-0,55	0,018313065
hsa-miR-25	12517,64	7639,53	1,64	0,49	0,018313065
hsa-let-7e	1297,51	2947,83	0,44	-0,82	0,019691385
hsa-let-7c	5660,31	8969,72	0,63	-0,46	0,019946719
hsa-let-7f	5382,15	9746,17	0,55	-0,59	0,019992878
hsa-let-7a	6795,89	12517,64	0,54	-0,61	0,022999135
hsa-let-7g	3428,42	6795,89	0,5	-0,68	0,023591848
hsa-miR-140-3p	9312,5	4621,29	2,02	0,7	0,024644601
hsa-miR-339-5p	312,11	12,44	25,08	3,22	0,025550318
hsa-miR-361-5p	606,46	53	11,44	2,44	0,030845953
hsa-miR-1283	2,33	22,22	0,11	-2,25	0,03712987
hsa-miR-18a*	1040,44	119,89	8,68	2,16	0,04393972
hsa-miR-26b	1085,22	2058,85	0,53	-0,64	0,044122011

## Discussion

While complex miRNA expression patterns have been reported for a huge variety of human tumors, far less information is available on miRNA expression patterns of blood cells derived from tumor patients. In the following we relate our miRNA expression profiling to both the miRNA expression in blood cells and in cancer cells of non-small cell lung cancer patients. We found significant down-regulation of hsa-miR-126 that was recently detected in blood cells of healthy individuals, but not in blood cells of lung cancer patients [10]. Down-regulation of hsa-miR-126 was also found in lung cancer tissue [25]. Functional studies on hsa-miR-126 revealed this miRNA as a regulator of the endothelial expression of vascular cell adhesion molecule 1 (VCAM-1), which is an intercellular adhesion molecule expressed by endothelial cells [26]. hsa-miR-126 is also reported to be an inhibitor of cell invasion in non-small cell lung cancer cell lines, and down-regulation of this miRNA might be a mechanism of lung cancer cells to evade these inhibitory effects [27]. Members of the hsa-let-7 family that were found down-regulated in our study were the first miRNAs reported as de-regulated in lung cancer [28]. This down-regulation of the let-7 family in lung cancer was confirmed by several independent studies [29-33]. Our data are also in agreement with a recent study showing the down-regulation of hsa-let-7a, hsa-let-7d, hsa-let-7f, hsa-let-7g, and hsa-let-7i in blood cells of lung cancer patients [10]. Notably, down-regulation of let-7 in lung cancer was strongly associated with poor clinical outcome [29]. The let-7 family members negatively regulate oncogene RAS [28]. The miRNA hsa-miR-22 that showed a high MI value and up-regulation in our study, was recently also reported to be up-regulated in blood cells of lung cancer patients [10]. The miRNA hsa-miR-19a that also showed a high MI value and up-regulation in our study was reported to be up-regulated in lung cancer tissue [34,35]. In contrast, hsa-miR-20a, which is significantly down-regulated in our experiments, was reported as up-regulated in lung cancer tissue [34,35]. The up-regulation of hsa-miR-20a was found in small-cell lung cancer cell lines, our study investigated only NSCLC. In summary, there is a high degree of consistency between miRNA expression found in the peripheral blood cells of lung cancer patients and miRNA expression in lung cancer tissue [25,29-37].

Some of the deregulated miRNAs identified in our study are also reported as de-regulated in other cancer entities, e.g. hsa-miR-346 in gastritic cancer, hsa-miR-145 in bladder cancer, and hsa-miR-19a in hepatocellular carcinoma and B-cell leukemia [38-42]. We also found miRNAs with high diagnostic potential e.g. high MI value, that were not yet related to cancer as for example hsa-miR-527 or hsa-mir-361-5p that were both up-regulated in blood cells of lung cancer patients.

Besides the deregulation of single miRNAs, we analyzed the overall expression pattern of miRNAs in peripheral blood cells of lung cancer patients in comparison to the pattern in blood cells of healthy controls. Recently, Chen et al. [10] reported a high correlation of 0.9205 between miRNA profiles in serum and miRNA profiles in blood cells, both in healthy individuals. The correlation of the miRNA profiles between serum and blood cells in lung cancer patients were significantly lower (0.4492). These results are indicative of deregulated miRNAs in blood and/or serum of patients and are in agreement with our data that show the deregulation of miRNAs in the blood cells of lung carcinoma patients. These deregulated miRNAs can be used to differentiate patients with lung cancer from normal controls with high specificity and sensitivity.

## Conclusion

This is the first evidence for the diagnostic potential of miRNA expression profiles in peripheral blood cells of cancer patients and healthy individuals. It remains to be seen whether other cancers and other non-cancer diseases also show a specific miRNA expression pattern that might be used to tell these diseases apart form controls and possibly apart from each other.

We are optimistic that the high specificity and sensitivity of the miRNAs deregulated in blood cells of lung carcinoma patients together with the high reproducibility of the applied technique will open avenues for applying this approach in prospective trials of a non-invasive diagnostic test.

## List of abbreviations

miRNA: microRNA; NSCLC: non-small cell lung cancer; qRT-PCR: quantitative real time polymerase chain reaction; MI: Mutual Information; GRTA: Geniom Realtime Analyzer; MPEA: microfluidic-based enzymatic on-chip labeling of miRNAs; SVM: Support Vector Machines.

## Competing interests

AK: salary (febit), patent (febit); AB salary (febit), patent (febit); MS salary (febit); FW salary (febit); AW salary (febit);

## Authors' contributions

AK contributed to study design, carried out the statistical analyses, initiated the study and contributed in paper writing. PL developed the protocol for miRNA extraction, extracted the miRNAs from blood cells, and contributed in paper writing. AB performed the miRNA screening. AW added the data into the miRNA database and contributed in the statistical evaluation. FW assisted the evaluation of the miRNA microarrays. MS contributed to study design and writing of the paper. HH contributed in study design, collected the blood samples and contributed in paper writing. HPL contributed in the bio-statistical analysis. EM contributed in study design, development of miRNA extraction, supervised the study and contributed in paper writing. All authors read and approved the final manuscript.

## Pre-publication history

The pre-publication history for this paper can be accessed here:

http://www.biomedcentral.com/1471-2407/9/353/prepub

## Supplementary Material

Additional file 1 **Table S1**. Details of all miRNAs for the miRVANA screening (columns A-I), MPEA Assay (columns K-S) and the MI (columns U-V). Significant miRNAs are colored. Click here for file

Additional file 2 **Table S2**. Mutual Information of biomarkers. This table contains for each miRNA biomarker the Mutual Information (MI) together with the enrichment as compared to the background MI. The miRNAs with MI higher than the highest background MI are colored red, the miRNAs with MI values higher than highest 5% background MI's in blue. Click here for file
